# Ozoile Reduces the LPS-Induced Inflammatory Response in Colonic Epithelial Cells and THP-1 Monocytes

**DOI:** 10.3390/cimb45020087

**Published:** 2023-02-05

**Authors:** Maria Paola Bertuccio, Valentina Rizzo, Salvatore Arena, Alessandra Trainito, Angela Simona Montalto, Daniela Caccamo, Monica Currò, Carmelo Romeo, Pietro Impellizzeri

**Affiliations:** 1 Department of Biomedical and Dental Sciences and Morpho-Functional Imaging, University of Messina, 98125 Messina, Italy; 2 Unit of Pediatric Surgery, Department of Human Pathology of Adult and Childhood “Gaetano Barresi”, University of Messina, 98125 Messina, Italy

**Keywords:** cytokines, inflammation, intestinal epithelial cells, monocytes, Ozoile, stable ozonides

## Abstract

Inappropriate activation of immune functions in intestinal epithelial cells can lead to inflammation that is characterized also by infiltration into intestinal tissue of monocytes/macrophages. Current therapies for intestinal inflammation include anti-inflammatory, immunosuppressive and biological drugs. Ozoile (stable ozonides) has been reported to exert anti-inflammatory effects. However, ozonated oil has been used mainly for topical applications and no data are available about its effects on intestinal cells or immune cells. In this study, we evaluated Ozoile effects on human HT-29 colonic cells and THP-1 monocytic cells stimulated with LPS to induce inflammation. HT-29 and THP-1 cells were treated with LPS in the presence/absence of Ozoile for 4 h. Biomarkers of inflammation, some members of tight junctions and the adhesion molecule ICAM were assessed by qRT-PCR. Protein expression was analyzed by Western blotting. The release of TNF-α and IL-1β was measured by ELISA. In HT-29, Ozoile inhibited LPS-induced expression of TNF-α, IL-1β, ZO-1, CLDN1, NOS2 and MMP-2 and increased the expression of Nrf2 and SOD2 antioxidant proteins. In THP-1 cells, the LPS induction of TNF-α, IL-1β and ICAM was counteracted by Ozoile treatment. Our in vitro results demonstrate the effectiveness of Ozoile in reducing the inflammatory response in intestinal and monocytic cells. Further in vivo studies are necessary to confirm its possible use for intestinal inflammatory conditions.

## 1. Introduction

Intestinal epithelial cells represent the primary protection from bacteria and many food metabolites and produce mediators, including cytokines and chemokines, contributing to the maintenance of intestinal homeostasis. Inappropriate activation of the immune functions of intestinal epithelial cells can lead to the establishment of intestinal inflammatory conditions, such as colitis, mucositis and inflammatory bowel diseases (IBD), characterized also by the infiltration into intestinal tissue of monocytes/macrophages [1].

IBD are mainly divided into ulcerative colitis (UC) and Crohn’s disease (CD), both of which cause digestive disorders and inflammation in the gastrointestinal tract. These diseases occur mainly in adults but in about 30% of the cases are diagnosed in children of all ages [2]. Typical symptoms of IBD are severe diarrhea, intestinal bleeding, abdominal pain, fatigue and weight loss, and they can have a progressive course leading to complications including stenosis and fistulas [3]. Extraintestinal manifestations of the disease such as growth failure, weight loss, anemia, joint symptoms and delayed puberty are frequently observed in children [4]. Patients with IBD need long-term therapeutic treatment, which can require the combination of several drugs. The most used include anti-inflammatory drugs such as immunosuppressive, corticosteroids, biological drugs and antibiotics; however, these are burdened by drug–drug interactions and various side effects, such as infections, lymphoma and skin cancer, as well as metabolic and cardiovascular diseases [5,6].

Over the last years clinical studies evaluating the possible use of complementary and alternative medicine, including herbal medicine and dietary supplementation (probiotics, prebiotics or fish oil), for the treatment of intestinal inflammatory diseases have shown encouraging results [7]. Although clinical trials results are not conclusive, maybe because of the heterogeneity of the study design and the used doses, it can be affirmed that omega-3, probiotics, parabiotics, postbiotics, prebiotics and synbiotics have potential benefits in different inflammatory diseases. For example, omega 3 fatty acid decreases the synthesis of inflammatory mediators by activating peroxisome proliferator-activated receptor gamma (PPAR)-γ and decreasing nuclear factor kappa-light-chain-enhancer of activated B cells (NF-κB) translocation to the nucleus, whereas probiotics and prebiotics in the gut are able to stimulate mucous secretion and proliferation of enterocytes [8].

Among alternative tools, the use of ozone therapy has been shown to be effective for the management of several disease [9]. Ozone is an unstable gaseous molecule that can be stabilized and used in various forms, such as ozonated water and ozonated oil [10,11]. The latter has the advantage of combining the biological activities of both ozone and olive oil, which has a high content of polyphenolic compounds and monounsaturated free fatty acids [12].

Beyond the first identified antimicrobial effects, ozone has been widely used for its properties to improve wound healing and modulate the immune system [13,14], providing effective therapy for inflammatory and infectious skin diseases [15].

Recently, we showed the effectiveness of Ozoile cream, which contains ozonated olive oil with vitamin E acetate, in the treatment of balanitis xerotica obliterans (BXO), a chronic inflammatory disease of the foreskin [16], also demonstrating a comparable effect with respect to corticosteroid therapy [17].

At present, therapies based on ozonated oil formulations have been used mainly for topical applications [18], and several medicinal and cosmetic products based on ozonated vegetable oils for the treatment of acne, herpes, psoriasis, fungal infections, bed sores and wounds are commercially available [18].

Recently, an in vivo study demonstrated that the addition of ozonated olive oil to dietary composition inhibits triglyceride accumulation in the liver and suppresses inflammatory factors alleviating hepatic steatosis in rats [19]. However, no data have been reported about the possible anti-inflammatory effects of ozonated oil in epithelial intestinal cells or immune cells, which are involved in the initiation and maintenance of inflammation leading to intestinal tissue damage.

Therefore, in this study we aimed to verify the effects of Ozoile on two different cell lines, the human colonic epithelial cell line HT-29 and the monocytic cell line THP-1, stimulated with lipopolysaccharide (LPS) to induce an inflammatory response [20,21,22].

## 2. Materials and Methods

### 2.1. Materials

HT-29 (HTB-38) and THP-1 (TIB-202) cells were purchased from the American Type Culture Collection (ATCC). RPMI-1640, D-MEM, fetal bovine serum (FBS), L-glutamine, HEPES, sodium pyruvate, glucose, 2-mercaptoethanol, penicillin/streptomycin mixture, phosphate buffered saline solution (PBS), mouse monoclonal antibody against β-actin, horseradish peroxidase (HRP)-conjugated anti-mouse and anti-rabbit secondary antibodies and other chemicals of analytical grade were from Sigma-Aldrich (Milan, Italy). Lipopolysaccharide (LPS) was from InvivoGen (San Diego, CA, USA). Rabbit monoclonal antibody for nuclear factor erythroid 2-related factor 2 (Nrf2) was from Bioss Antibodies (Aurogene Srl, Rome, Italy). The rabbit antibodies against NOS2 and laminin B1 were purchased from Abcam (Cambridge, UK). Mouse MMP-2 polyclonal antibody was obtained from Chemicon International Inc. (Merck, Milan, Italy). Mouse monoclonal antibodies against SOD2 were from Santa Cruz Biotechnology (D.B.A. Italia S.R.L., Milan, Italy). Pierce ECL Western Blotting Substrate, TRIzol, the high-capacity cDNA archive kit and SYBR Select Master mix were from Life Technologies (Milan, Italy). Specific primers for real-time PCR were from Eurofins Genomics (Ebersberg, Germany). ELISA kits for the quantitative detection of human IL-1β and TNF-α were from Cloud-Clone Corp. (Katy, TX, USA).

### 2.2. Ozoile Preparation

Ozoile was kindly provided by Erbagil s.r.l. (Benevento, Italy). Ozoile is a pool of stable ozonide molecules produced in a patented process by 1,3 dipolar cycloaddition of ozone, obtained from pure oxygen through an electrical discharge of 10,000 volts, with olephinic bonds from the olive oil according to Criegee mechanism. The first step reaction leads to the formation of molozonide, also known as Criegee intermediate, which transposes into ozonides by the combination of a carbonyl fragment and a zwitterionic species. In this patented Erbagil process stable ozonides are produced.

Based on previous tests carried out at ABICH laboratories (Verbania, Italy), which performed preliminary studies to evaluate the cytotoxicity of the product, Ozoile was dissolved in isopropanol instead of DMSO to favor the dispersion of the oil to be tested. As recently reported [23], isopropanol concentrations up to 1% did not show cytotoxic effects. According to these results, our preliminary experiments that aimed to evaluate isopropanol’s effects on cell viability and cytokine production showed that there was no difference between cells treated with 1% isopropanol and control cells. Thus, we used a stock solution (200 mg Ozoile/mL isopropanol) that was diluted to different concentrations with PBS and then added to the culture medium in a range of concentration from 0.1 to 2 mg/mL. During all procedures, Ozoile solutions were stirred continuously to avoid phase separation. In all experiments, equal volumes of PBS/isopropanol were added to the medium of control cultures.

### 2.3. Cell Culture and Treatment

THP-1 cells were grown in RPMI 1640 supplemented with L-glutamine (2 mM), HEPES (10 mM), sodium pyruvate (1 mM), glucose (2.5 g/L), 2-mercaptoethanol (0.05 mM), 10% heat-inactivated fetal bovine serum (FBS) and 1% penicillin/streptomycin, whereas HT-29 cells were grown in D-MEM supplemented with L-glutamine (2 mM), 1% penicillin/streptomycin and 10% heat-inactivated fetal bovine serum (FBS); both cell lines were maintained at 37 °C in a 5% CO_2_/95% air humidified atmosphere. Medium was renewed every 2 days and splits were performed when cells reached maximum density. In our experimental conditions, cells were seeded at a density of 3 × 10^5^ cells/mL into culture plates in complete medium plus 10% FBS and incubated at 37 °C. Prior to stimulation with LPS and Ozoile, HT-29 and THP-1 cells were seeded into culture plates in RPMI plus 5% fetal bovine serum and incubated for 24 h at 37 °C to induce cell synchronization. Sub-confluent cells were treated with LPS (1 µg/mL) for 4 h in the presence or absence of Ozoile (0.1–0.5–1–2 mg/mL), which was added to the culture medium 30 min prior to LPS treatment.

As reported in our previous study [24], preliminary dose-response and time-dependent experiments were carried out to choose the LPS concentration and incubation time in HT-29 cells. The incubation with LPS (1 μg/mL) for 4 h evoked the most significant increases in cytokine mRNA levels. The same conditions were used to stimulate THP-1 cells, and this demonstrated similar effects.

Preliminary optimization studies were also performed to choose Ozoile concentrations.

### 2.4. Cell Viability Assay

Cell viability was measured by a quantitative colorimetric assay with 3-[4,5-dimethylthiazol-2-yl]-2,5-diphenyltetrazolium bromide (MTT). For this assay, HT-29 cells were cultured in 96-well culture plates. After treatment, the cells were washed and incubated with fresh medium containing MTT (0.5 mg/mL) at 37 °C for 4 h. Then, insoluble formazan crystals were dissolved in 100 µL of acidic isopropanol (0.04 N HCl in absolute isopropanol) at 37 °C for 1 h. The optical density in each well was evaluated by spectrophotometrical measurement of the absorbance at 570 nm using a microplate reader (Tecan Italia, Cologno Monzese, Italy). The percentage cell viability was calculated as (the OD of treated sample/the OD of untreated sample) × 100. The cell viability of THP-1 cells was determined as previously reported [25]. Briefly, after treatment, THP-1 cells were harvested by centrifugation and incubated in 96-well culture plates at a density of 5 × 10^4^ cells/well with fresh red-phenol free medium containing MTT (0.5 mg/mL) at 37 °C for 4 h. Then, 100 µL of a 0.04 N HCl/isopropanol solution was added to each well and after 1 h the absorbance at 570 nm was read using a microplate reader. All experiments were performed in quintuplicate.

### 2.5. Gene Expression Analysis by Real-Time PCR

The mRNA levels of IL-1β, TNF-α, IL-4, NOS2, MMP2, ZO1, CLDN1 and ICAM were assessed by real-time PCR using SYBR-green-based gene expression analysis. β-Actin was used as the endogenous control. The primer sequences used are reported in Table 1. Cells were seeded at a density of 3 × 10^5^ cells/mL into six-well culture plates and, after treatment, 2 µg of RNA isolated with TRIzol reagent was reverse transcribed using a High-Capacity cDNA Archive kit according to the manufacturer’s instructions.

Quantitative PCR reactions were set up in triplicate in a 96-well plate and carried out in a final volume of 20 µL that contained 1× SYBR green PCR Mastermix, 0.1 µM specific primers and 25 ng cDNA. SYBR Green real-time qRT-PCR was performed in a 7900HT Fast Real-Time PCR System (Applied Biosystems, Foster City, CA, USA) with the following profile: one cycle at 95 °C for 10 min, followed by 40 cycles at 95 °C for 15 s and 60 °C for 1 min. A standard dissociation stage was added to assess primer annealing specificity. Data were collected and analyzed using SDS 2.3 and RQ manager 1.2 software (Applied Biosystems, Foster City, CA, USA) and the 2^(−ΔΔCt)^ relative quantification method. Values are presented as fold change relative to untreated cells.

### 2.6. Western Blotting

Protein expression of NOS2, MMP2 and SOD2 in HT-29 cells was evaluated by Western blot analyses. For this assay, cells were seeded at a density of 3 × 10^5^ cells/mL into six-well culture plates. After treatment, cells were lysed using ice-cold RIPA buffer supplemented with Protease Inhibitor Cocktail (SIGMA Aldrich, Milan, Italy) and cell debris were removed by centrifugation at 8000× *g* at 4 °C for 20 min. Protein concentrations were determined by the Bradford method and equal amounts of protein (30 µg) were separated by 10% SDS-polyacrylamide gel electrophoresis and transferred to nitrocellulose membranes. After that, membranes were incubated for 1 h at room temperature in Tris-buffered saline containing 0.15% Tween 20 (TBS-T) with 5% non-fat dry milk; then, membranes were incubated over-night at 4 °C with primary antibodies against NOS2, MMP2, SOD2 (diluted 1:500 in TBS-T) or β-actin (diluted 1:6000). Afterwards, the membranes were incubated with horseradish-peroxidase-conjugated anti-mouse or -rabbit secondary antibodies (diluted 1:10,000 and 1:3000, respectively).

The presence of Nrf2 in the nuclear compartment of HT-29 cells was also detected by Western blot analysis. Membranes were probed with primary antibodies against Nrf2 (diluted 1:500) and laminin B1 (diluted 1:1000), followed by incubation with horseradish-peroxidase-conjugated anti-rabbit secondary antibody (diluted 1:3000).

Immunoblots were developed by ECL chemiluminescent detection system kit and the bands were scanned and quantified by scanning densitometry using a bio-image analysis system (C-DiGit, Li-cor, Lincoln, NE, USA).

### 2.7. Evaluation of Cytokine Secretion by ELISA

The release of TNF-α and IL-1β cytokines in cell-free culture supernatants of HT-29 and THP-1 cells was measured using enzyme linked immunosorbent assay (ELISA) kits according to the manufacturer’s guidelines. Supernatants recovered from treated and untreated cells, grown in six-well culture plates, were concentrated 10-fold by freeze-drying, and then the samples were reconstituted by the addition of distilled water. To detect the cytokines, 100 µL of standards or samples was incubated in 96-well plates for 1 h at 37 °C. Then, 100 µL of Detection Reagent A was added and the plate incubated for a further 1 h at 37 °C. After washing 3 times with 350 µL of Wash Buffer, 100 µL of Detection Reagent B was added and the plate incubated for 30 min at 37 °C. After washing 5 times, 90 µL of the substrate solution was added to each well and the plates were incubated in the dark for 15 min at 37 °C. The enzyme reaction was then stopped by pipetting 50 µL of stop solution into each well; the absorbance was determined at 450 nm using a microplate reader (Tecan, Milan, Italy). All experiments were performed in triplicate.

### 2.8. Statistical Analysis

Data obtained from five separate experiments are expressed as mean ± SEM and were analyzed by one-way analysis of variance (ANOVA) followed by the Bonferroni post hoc test using Graph Pad Prism software (version 5.00) (San Diego, CA, USA). *p* values lower than 0.05 were considered significant.

## 3. Results

**Figure 1:**
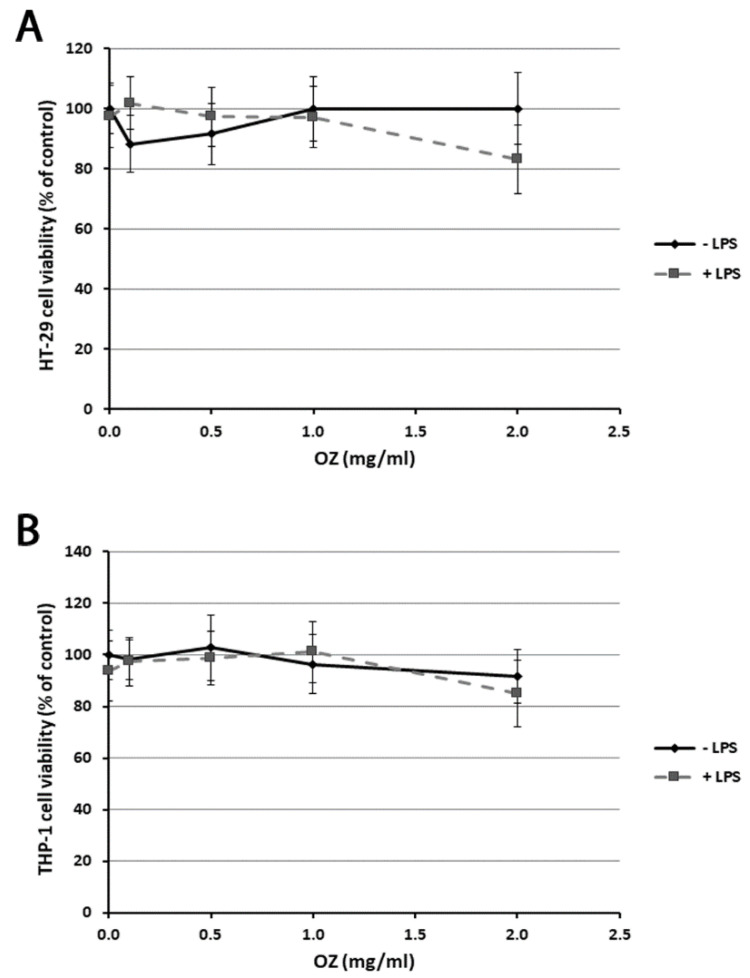
Effects of Ozoile (OZ) on HT-29 (**A**) and THP-1 (**B**) cell viability in the presence or absence of LPS (1 µg/mL). Different concentrations of Ozoile (0.1, 0.5, 1 and 2 mg/mL) were added to the culture medium 30 min before LPS treatment (1 µg/mL for 4 h), and then cell viability was assessed by the MTT test. Results are expressed as percentages relative to untreated cells. Data are means ± SEM from five independent experiments.

In order to assess Ozoile toxicity in cell cultures, preliminary experiments were carried out using an MTT test. The exposure of HT-29 and THP-1 cells for 4 h to different Ozoile concentrations (in the range 0.1–2 mg/mL), both in presence and absence of LPS, did not cause any significant variation in cell viability in comparison to control cells. Only a slight but not significant reduction in cell viability was observed in HT-29 cells treated with 0.1 or 0.5 mg/mL of Ozoile in absence of LPS. Instead, a reduction of about 18% in cell viability was observed in cells treated with LPS and 2 mg/mL Ozoile (Figure 1A,B). Thus, in the subsequent experiments we used Ozoile in the concentration range of 0.1–1 mg/mL.

**Figure 2:**
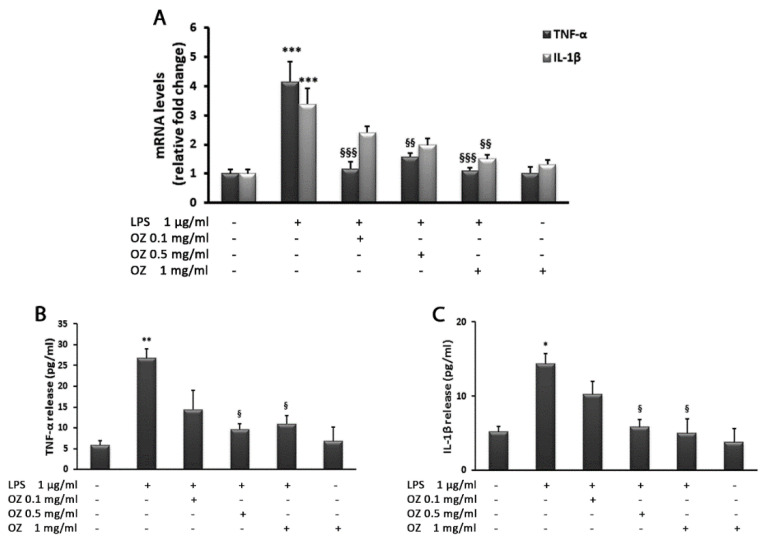
Effects of Ozoile (OZ) on cytokine gene expression (**A**) and release (**B**,**C**) in HT-29 cells stimulated with LPS. (**A**) HT-29 cells were treated with different concentrations of Ozoile (0.1, 0.5 and 1 mg/mL) 30 min before LPS treatment (1 µg/mL for 4 h). The mRNA transcript levels of TNF-α and IL-1β were analyzed by real-time PCR. (**B**,**C**) Cytokine release into culture medium was measured by ELISA. Data are expressed as means ± SEM from three independent experiments. * *p* < 0.05, ** *p* < 0.01 and *** *p* < 0.001 indicate significant differences vs. control cells. ^§^ *p* < 0.05, ^§§^ *p* < 0.01 and ^§§§^ *p* < 0.001 indicate significant differences vs. LPS-treated cells.

To evaluate the LPS-induced inflammatory response, we evaluated the expression and release of cytokines. In HT-29 cells, TNF-α and IL-1β transcript levels analyzed by real-time PCR showed a significant increase after stimulation with LPS (1 μg/mL) in comparison with unstimulated cells. In particular, TNF-α increased 4.1-fold and IL-1β increased 3.4-fold. The inflammatory response induced by LPS was effectively counteracted by Ozoile treatment. A significant reduction in proinflammatory cytokine expression (TNF-α and IL-1β) was found in cells treated with Ozoile compared with cells exposed to LPS alone. The treatment with different concentrations of Ozoile (0.1–0.5–1 mg/mL) restored TNF-α levels to those found in control cells (Figure 2A). Ozoile addition to LPS-treated HT-29 cells also induced a reduction in TNF-α release into the culture medium in comparison to LPS-stimulated cells (Figure 2B). Regarding IL-1β expression, Ozoile treatment induced a dose-dependent decrease; in particular, at the highest Ozoile concentration (1 mg/mL), the IL-1β mRNA levels were reduced by approximately 55% in comparison with LPS-treated cells (Figure 2A). Similarly, Ozoile exposure decreased IL-1β release into the culture medium in comparison with LPS-stimulated cells (Figure 2C). Cell treatment with Ozoile (1 mg/mL) alone did not cause significant changes in the production of both the analyzed cytokines in respect to control cells (Figure 2A–C).

**Figure 3:**
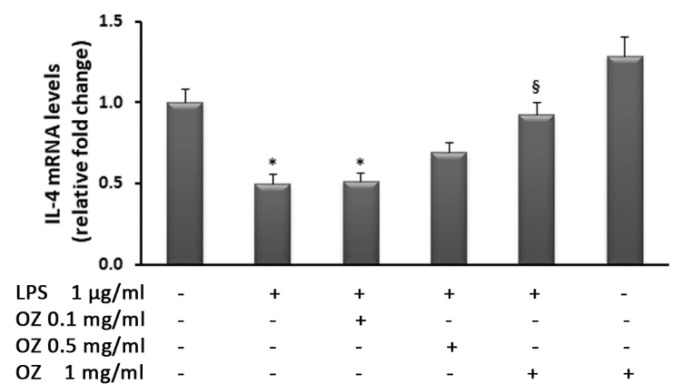
Effects of Ozoile (OZ) on IL-4 gene expression in HT-29 cells stimulated with LPS. HT-29 cells were treated with different concentrations of Ozoile (0.1, 0.5 and 1 mg/mL) 30 min before LPS exposure (1 µg/mL for 4 h). The mRNA transcript levels were analyzed by real-time PCR. Data are expressed as means ± SEM from three independent experiments. * *p* < 0.05 indicates a significant difference vs. control cells. ^§^ *p* < 0.05 indicates a significant difference vs. LPS-treated cells.

Further results supporting the efficacy of Ozoile to counteract inflammation have been obtained by the analysis of the anti-inflammatory cytokine IL-4. Indeed, Ozoile restored IL-4 transcript levels that were down-regulated by LPS (Figure 3). In particular, the highest Ozoile concentration was able to re-establish the basal levels of IL-4 gene expression.

**Figure 4:**
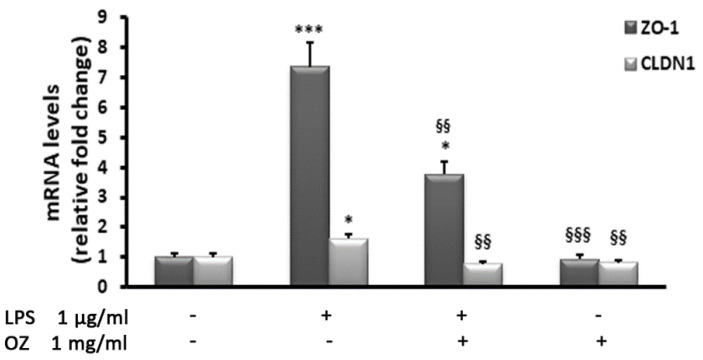
Effects of Ozoile (OZ) on ZO-1 and CLDN1 gene expression in HT-29 cells stimulated with LPS. HT-29 cells were treated with 1 mg/mL of Ozoile 30 min before LPS treatment (1 µg/mL for 4 h). The mRNA transcript levels were analyzed by real-time PCR. Data are expressed as means ± SEM from three independent experiments. * *p* < 0.05 and *** *p* < 0.001 indicate significant differences vs. control cells. ^§§^ *p* < 0.01 and ^§§§^ *p* <0.001 indicate significant differences vs. LPS-treated cells.

In HT-29 cells, we also examined the transcript levels of the tight junction (TJ) members zonulin 1 (ZO-1) and claudin 1 (CLDN1), whose dysregulation contributes to the development of IBD [26]. As shown in Figure 4, LPS treatment produced a remarkable increase in the mRNA levels of ZO-1 and a slight but significant increase in CLDN1 transcript amounts. These effects were reduced by the addition of Ozoile.

**Figure 5:**
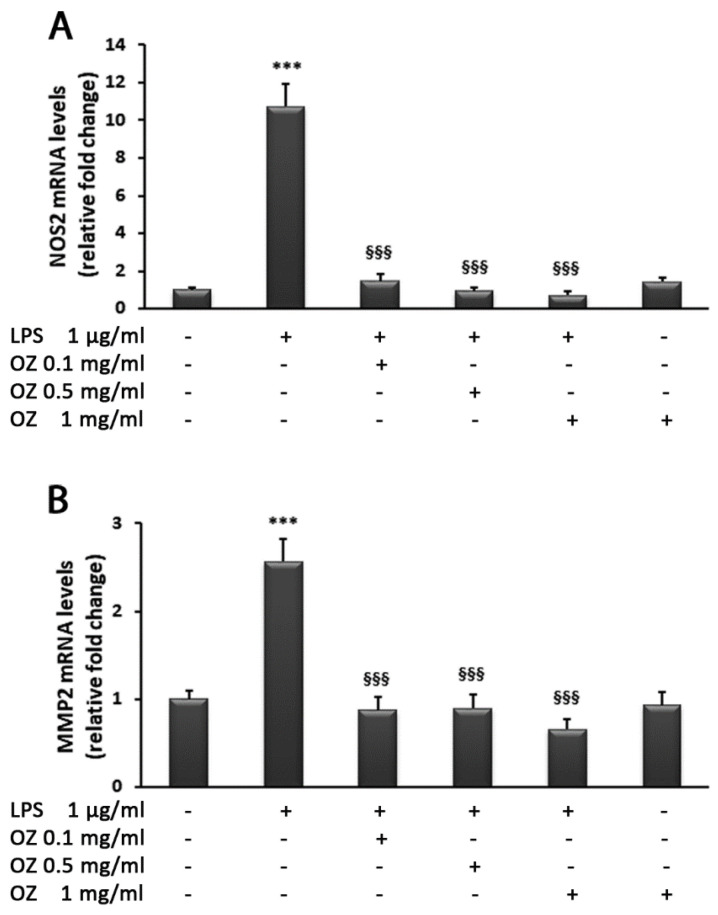
Effects of Ozoile (OZ) on NOS2 (**A**) and MMP2 (**B**) gene expression in HT-29 cells stimulated with LPS. HT-29 cells were treated with different concentrations of Ozoile (0.1, 0.5 and 1 mg/mL) 30 min before LPS treatment (1 µg/mL for 4 h). The mRNA transcript levels of NOS2 and MMP2 were analyzed by real-time PCR. Data are expressed as means ± SEM from three independent experiments. *** *p* < 0.001 indicates a significant difference vs. control cells. ^§§§^ *p* < 0.001 indicates a significant difference vs. LPS-treated cells.

Further, the effects of Ozoile on the expression of NOS2 and MMP2 were evaluated. In comparison with untreated cells, LPS treatment induced the up-regulation of NOS2 mRNA levels 10-fold; this effect was significantly reduced by Ozoile at all concentrations (0.1–0.5–1 mg/mL) (Figure 5A). Similar results were observed for the MMP2 transcript levels, although LPS induced an increase of only 2.5-fold (Figure 5B). In cells treated with Ozoile in absence of LPS stimulation, no evident changes were found in comparison to untreated cells.

**Figure 6:**
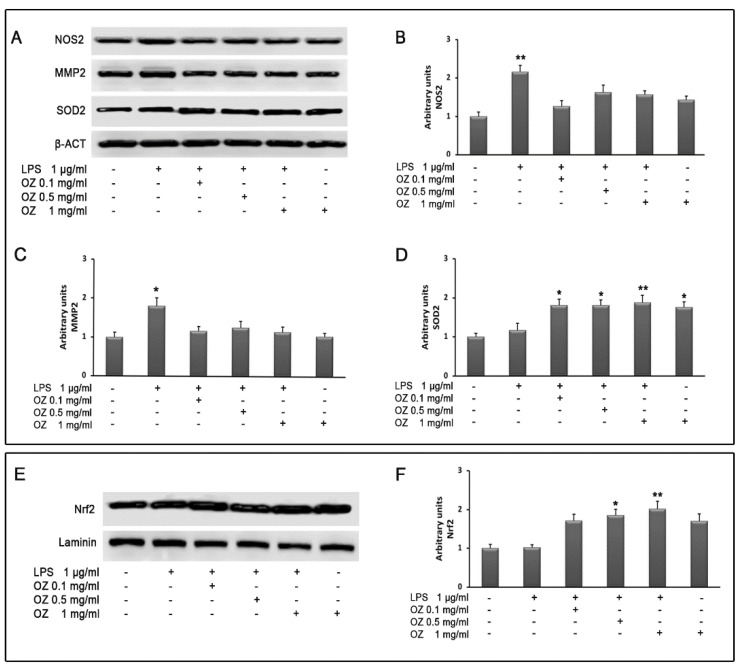
Evaluation of NOS2, MMP2, SOD2 (**A**) and Nrf2 (**E**) protein expression by Western blotting in HT-29 cells treated with different concentrations of Ozoile (0.1, 0.5 and 1 mg/mL), added 30 min before LPS treatment (1 µg/mL for 4 h). Densitometric analysis of NOS2 (**B**), MMP2 (**C**) and SOD2 (**D**) after normalization against β-actin and of Nrf2 (**F**) after normalization against laminin. The results are expressed as means ± SEM from three independent experiments. * *p* < 0.05 and ** *p* < 0.01 indicate significant differences vs. control cells.

Western blot analysis confirmed real-time PCR results by showing an increase in NOS2 and MMP2 protein expression in LPS-treated cells that was suppressed by Ozoile treatment at all concentrations (0.1–0.5–1 mg/mL) (Figure 6A–C).

To characterize the possible pathway activated in response to Ozoile in HT-29 cells, we analyzed the expression of the transcription factor Nrf2, which regulates the expression of antioxidant enzymes. Cell incubation with LPS did not cause any variation in Nrf2 expression compared with controls (Figure 6E,F), whereas Ozoile both in presence or absence of LPS increased Nrf2 protein levels. A similar trend was observed for the expression of SOD2, which is a target of Nrf2 (Figure 6A,D).

**Figure 7:**
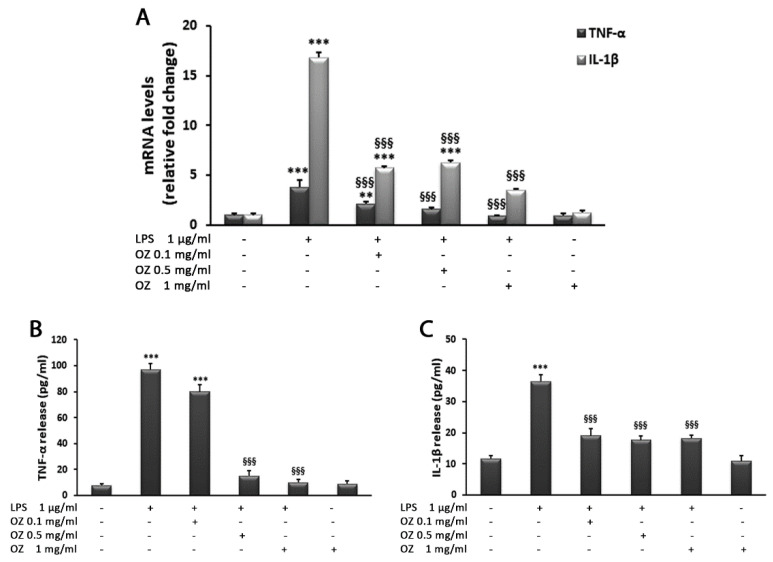
Effects of Ozoile (OZ) on TNF-α and IL-1β gene expression (**A**) and release (**B**,**C**) in THP-1 cells stimulated with LPS. (**A**) THP-1 cells were treated with different concentrations of Ozoile (0.1, 0.5 and 1 mg/mL) before LPS treatment (1 µg/mL for 4 h). The mRNA transcript levels were analyzed by real-time PCR. (**B**,**C**) Cytokine release into the culture medium was measured by ELISA. Data are expressed as means ± SEM from three independent experiments. ** *p* < 0.01 and *** *p* < 0.001 indicate significant differences vs. control cells. ^§§§^ *p* < 0.001 indicates a significant difference vs. LPS-treated cells.

The expression levels of proinflammatory cytokines have also been studied in the human monocytic cell line THP-1. The analysis showed that LPS stimulation of THP-1 cells resulted in a dramatic increase in TNF-α and IL-1β mRNA when compared with control cells (Figure 7A). In particular, the increase in IL-1β transcript levels was higher than that observed for TNF-α. Ozoile was able to reduce these effects. Indeed, the treatment with Ozoile at concentrations of 0.5 and 1 mg/mL decreased TNF-α levels to those similar to control cells, whereas the lowest Ozoile concentration (0.1 mg/mL) did not restored the basal levels even if it significantly reduced the LPS-induced increase in TNF-α (Figure 6A). Also, Ozoile treatment at all concentrations significantly decreased the IL-1β transcript levels when compared with LPS-treated cells; the highest concentration (1 mg/mL) was more effective, reducing the IL-1β transcript levels by approximately 80% in comparison to LPS-treated cells (Figure 7A). Ozoile treatment in absence of LPS stimulation did not affect mRNA levels for both the analyzed cytokines, their levels being similar to those expressed in control cells (Figure 7A). The analysis of cytokines release into the culture medium confirmed the efficacy of Ozoile in counteracting the LPS proinflammatory effects (Figure 7B,C).

**Figure 8:**
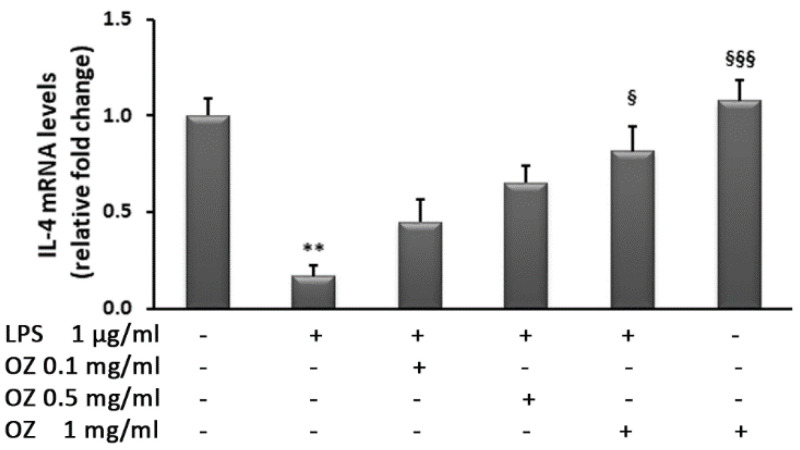
Effects of Ozoile (OZ) on IL-4 gene expression in THP-1 cells stimulated with LPS. THP-1 cells were treated with different concentrations of Ozoile (0.1, 0.5 and 1 mg/mL) 30 min before LPS exposure (1 µg/mL for 4 h). The mRNA transcript levels were analyzed by real-time PCR. Data are expressed as means ± SEM from three independent experiments. ** *p* < 0.01 indicates a significant difference vs. control cells. ^§^ *p* < 0.05 and ^§§§^ *p* < 0.001 indicate significant differences vs. LPS-treated cells.

THP-1 cell stimulation with LPS induced a drastic reduction in IL-4 mRNA levels that were restored by Ozoile treatment at the highest concentration (Figure 8).

**Figure 9:**
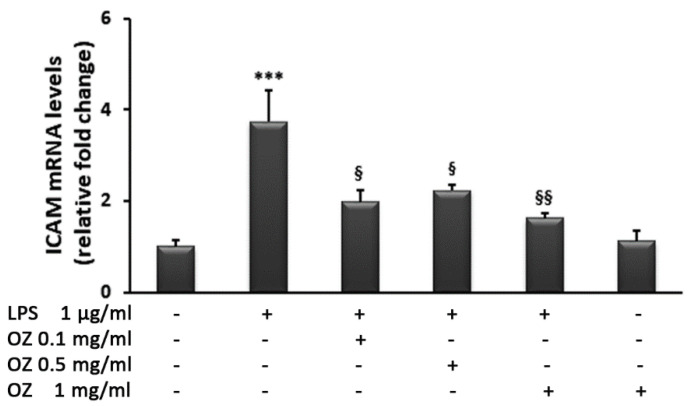
Effects of Ozoile (OZ) on ICAM gene expression in THP-1 cells stimulated with LPS. THP-1 cells were treated with different concentrations of Ozoile (0.1, 0.5 and 1 mg/mL) before LPS treatment (1 µg/mL for 4 h). The mRNA transcript levels were analyzed by real-time PCR. Data are expressed as means ± SEM from three independent experiments. *** *p* < 0.001 indicates a significant difference vs. control cells. ^§^ *p* < 0.05 and ^§§^ *p* < 0.01 indicate significant differences vs. LPS-treated cells.

To evaluate the effect of Ozoile on the extravasation of monocytic cells from blood to inflamed tissues, we assessed the expression of ICAM, an adhesion molecule involved in the process cited above, in THP-1 cells. As shown in Figure 9, LPS stimulation increased ICAM mRNA levels 3.7-fold in THP-1 monocytes, whereas Ozoile treatment reduced this effect. Indeed, incubation with LPS in the presence of Ozoile restored the expression levels of ICAM to values similar to those observed in control cells. The analysis of ICAM gene expression in cells incubated with Ozoile the in absence of LPS stimulation, showed no significant differences compared with control cells.

## 4. Discussion

The use of ozone therapy has yielded promising results in several medical applications, such infection and vascular and immune diseases [27]. The possibility to increase the stability of ozone producing ozonides by the reaction with the unsaturated fatty acid of vegetable oils has facilitated its use for pharmaceutical application [18]. Ozonated oils have been reported to be effective for the treatment of skin infections, ulcers and burns, as well as oral, ophthalmologic, joint and gynecological diseases [18,28,29]. In a previous study, we showed that preoperative treatment with Ozoile in children with BXO reduced the transcript levels of pro-inflammatory cytokines in treated foreskins. In addition, changes in the expression of E-cadherin and VEGF induced by Ozoile treatment suggested its role in the re-epithelialization process of foreskin affected by BXO, maybe by stimulating basement membrane reconstruction and angiogenesis [16,17].

Ozone therapy has been also proposed as an adjuvant therapy for the treatment of IBD with promising results [30]. It has been found that rectal ozone therapy associated with sulfasalazine treatment was more effective than sulfasalazine alone to relieve symptoms and improve the histological architecture in patients with ulcerative colitis [31].

In line with these results, in our in vitro study we demonstrated that Ozoile is able to reduce the production of the proinflammatory cytokines TNF-α and IL-1β, while increasing the expression of the anti-inflammatory cytokine IL-4 in HT-29 intestinal epithelial cells. Cytokines have a crucial role in the pathogenesis and progression of intestinal inflammation, being the controllers of intestinal homeostasis. The imbalance between pro- and anti-inflammatory cytokines hinders the resolution of inflammation and stimulates effector mechanisms of tissue destruction [32]. Thus, targeting the cytokine signaling pathway cascades permits the improvement of the outcome of patients with intestinal inflammation [33].

Cytokines can also lead to increased permeability of intestinal epithelial barrier, one of the hallmarks of IBD [34]. Paracellular permeability is regulated by a multitude of protein complexes, such as tight junctions (TJs), adherens junctions and gap junctions, which are essential for establishing cell–cell contact. Among TJs, zonulin is considered the master regulator of paracellular permeability, and elevated serum and fecal zonulin concentrations have been found in patients with IBD [35,36]. A recent in vivo study [37] also reported increases in zonulin gene expression in gut tissues associated with the alteration of cellular permeability, suggesting that these events represent an early step leading to the development of chronic inflammatory disease. Claudins are other membrane proteins that form selectively permeable barriers and are essential for transcellular and paracellular transport coupling. An increase in claudin-1 expression has been observed in colonocytes of patients with active IBD [38]. In addition, claudin-1 and claudin-2 were up-regulated in IBD-associated dysplasia and sporadic adenomas, as well as in colon carcinomas and metastatic lesions, suggesting their involvement in tumorigenesis and the invasiveness of colonic epithelial cells [38]. Our data, demonstrating the inhibitory action of Ozoile on both ZO-1 and CLDN1, support the hypothesis that Ozoile may have protective effects on intestinal inflammatory conditions and could also maybe reduce the risk of neoplastic transformation.

Proinflammatory cytokines are also involved in the up-regulation of NOS2. In the intestinal epithelium and inflamed colonic mucosa of IBD patients, it has been reported there is an increase in NOS2 expression [39]. Furthermore, a positive correlation between proinflammatory cytokine levels and the production of nitric oxide (NO) has been observed in patients with IBD [40]. NO is a free radical that exerts several physiological functions; however, the activation of NOS2 leads to an excessive production of NO that can cause an exacerbation of inflammation through the generation of peroxynitrite. This, in turn, can have cytotoxic effects by producing lipid peroxidation and DNA damage [41]. In line with these observations, the reduction in NOS2 gene expression observed in HT-29 cells treated with Ozoile indicates a possible protective role for Ozoile against cell damage secondary to nitrosative stress triggered by proinflammatory stimuli, such as LPS.

We also found that in HT-29 cells Ozoile treatment was able to reverse the LPS-induced increase of MMP2 gene expression. This gene has been shown to be up-regulated in a rat model of TNBS-induced colitis and was correlated with the severity of the disease [42]. On the other hand, some studies found increased levels of MMP2 in intestinal epithelium in IBD, also in the pediatric age [43], and hypothesized that in this context MMP2 may play a protective role by remodeling the extracellular matrix to repair tissue damage [44].

As recently demonstrated for low O3 concentrations [45], the moderate oxidative stress induced by Ozoile could stimulate a protective response via the activation of the Nrf2-mediated Keap1-dependent pathway, which controls the expression of antioxidant and detoxification genes [46,47] and ECM degradation proteins including matrix metalloproteinases (MMPs) [48,49]. The Nrf2/Keap1 axis plays an important role in the development of the gastrointestinal (GI) tract and the maintenance of its proper functionality. Further, it has been shown that the correct regulation of this axis is important in preventing inflammatory bowel disease (IBD) [50]. In vivo studies demonstrated that Nrf2 knockout mice were more prone to DSS-induced disease and exhibited higher levels of proinflammatory cytokines and lower expression of antioxidant enzymes in comparison with wild type mice.

Our data revealed that Ozoile is capable of increasing the expression of Nrf2 and SOD2, suggesting that Nrf2 can counteract ROS production and lower the accumulation of oxidative stress substances. According to these results, we can hypothesize that Ozoile may exert a beneficial effect in intestinal cells, modulating inflammation and nitrosative and oxidative stresses and leading to the inhibition of MMP2 expression and thus reducing cell alterations caused by LPS.

Given that monocyte recruitment and infiltration is a key feature of inflamed tissues, we evaluated the effects of Ozoile in monocytic THP-1 cells. Blood monocytes play a pivotal role in innate immune defense through several mechanisms including the phagocytosis of pathogens and the release of cytokines. During intestinal inflammation, monocytes are recruited into colon tissue and quickly differentiate into resident macrophages. Proinflammatory cytokines produced by monocytes/macrophages contribute to the disruption of the intestinal barrier function by altering the structure and function of tight junctions [51]. On the other hand, the anti-inflammatory cytokines, including IL-4, have been shown to suppress proinflammatory signaling, limiting immune cell accumulation in gut tissues [52,53].

Several members of the immunoglobulin superfamily, such as intercellular adhesion molecule-1 (ICAM-1) or CD54, vascular cell adhesion molecule-1 (VCAM-1) or CD106, and mucosal addressin cell adhesion molecule-1 (MAdCAM-1), have established pathogenetic roles in intestinal inflammation, acting as mediators of the aberrant trafficking of leukocytic cells to the inflamed mucosal sites [54]. Interference with molecules that mediate leukocyte traffic has already shown efficacy and been approved by the US Federal Drug Administration for use in Crohn’s disease [54].

In this study, we found that monocytic THP-1 cells exposed to LPS acquired an inflammatory phenotype, as shown by the increase in TNF-α and IL-1β production and expression of the adhesion molecule ICAM-1. Interestingly, these effects were reduced upon treatment with Ozoile, suggesting its ability to limit the response to inflammatory stimuli in immune cells.

Although our results are encouraging regarding the effectiveness of Ozoile in counteracting colonic cell inflammation, the impact of Ozoile on gut microbiota has not been evaluated. Thus, the effects of Ozoile on gut microbiota need further study, because Ozoile could both improve or worsen the healthy composition of microbiota due to its antimicrobial action. In this regard, ozone has been reported to activate immune and anti-inflammatory signaling with potential effects on gut microbiota [55]. Alterations to the gut microbiota are also the result of ozone-induced increases in adrenocorticotropic hormone and corticosterone levels through the modulation of hypothalamic–pituitary–adrenal axis. In particular, ozone has been reported to facilitate gut microbiome biotransformation, change microbiota diversity and abundance, disrupt intestinal epithelial tight junctions and stimulate immune cells and adrenergic neurons [55]. Moreover, ozone has been shown to increase various amino acids, polyamines and metabolites of gut microbiota, indicating gut microbiome alterations [56].

In conclusion, our preliminary in vitro results demonstrate the effectiveness of Ozoile in reducing the inflammatory response in colonic intestinal cells and monocytes. Although continuous cell lines are widely used in several fields of medical research, additional investigations could be carried out on primary human colonic cells and ex vivo isolated monocytes.

To verify the possible use of Ozoile as adjuvant therapy in patients with intestinal inflammatory conditions, further studies in animal models should be carried out.

## Figures and Tables

**Table 1:** Primers used for real-time PCR analysis of gene expression.

Target	Primer Sequence 5′ > 3′	
	Forward	Reverse
*TNF-α*	GTGAGGAGGACGAACATC	GAGCCAGAAGAGGTTGAG
*IL-1β*	GCTTATTACAGTGGCAATGA	TAGTGGTGGTCGGAGATT
*IL-4*	CCACGGACACAAGTGCGATAT	GGCAGCAAAGATGTCTGTTACG
*NOS2*	TGACCTCCTAACAAGTAGCA	CAGCAGCAAGTTCCATCT
*MMP2*	TGATCTTGACCAGAATACCATCGA	GGCTTGCGAGGGAAGAAGTT
*ZO1*	TCATCCCAAATAAGAACAGAGC	GAAGAACAACCCTTTCATAAGC
*CLDN1*	GTTGGGCTTCATTCTCGCCTT	CCTGGGCGGTCACGATGTTGTC
*ICAM*	CCCATGAAACCGAACACAC	ACTCTGTTCAGTGTGGCACC
*β-actin*	TTGTTACAGGAAGTCCCTTGCC	ATGCTATCACCTCCCCTGTGTG

## Data Availability

The data presented in this study are available on request from the corresponding author. The data are not publicly available due to stable ozonides are produced by a patented process.
